# Supplementary material for: Cytokine Receptor-like Factor 1 (CRLF1) and Its Role in Osteochondral Repair
Source: Cells. 2024 Apr 28;13(9):757. doi: 10.3390/cells13090757 (PMC11083199; doi:10.3390/cells13090757)
Supplement: Supplementary file 1 [file cells-13-00757-s001.zip › cells-2937063-supplementary.pdf]

Table S1. Sequences of Primers used for qPCR.

Gene		Primers (5' to 3')
Aggrecan	Forward<br>Reverse	TGCGGGTCAACAGTGCCTATC<br>CACGATGCCTTTCACCACGAC
CLC	Forward<br>Reverse	CCATGGACCTCCGAGCAG<br>GTCCCCTGTGCGATTGAGAG
CRLF1	Forward<br>Reverse	ATGAAGGACTTGACCTGCCG<br>TGTGTTGTCCTGGCCATACC
IL-6	Forward<br>Reverse	AGACAGCCACTCACCTCTTCAG<br>TTCTGCCAGTGCCTCTTTGCTG
Sox-9	Forward<br>Reverse	ACACACAGCTCACTCGACCTTG<br>AGGGAATTCTGGTTGCTCCTCT
COL2A1	Forward<br>Reverse	TTCAGCTATGGAGATGACAATC<br>AGAGTCCTAGAGTGACTGAG
COL10A1	Forward<br>Reverse	CCCTCTTGTTAGTGCCAACC<br>AGATTCCAGTCCTTGGGTCA
MMP-13	Forward<br>Reverse	GACTGGTAATGGCATCAAGGGA<br>CACCGGCAAAAGCCACTTTA
18S	Forward<br>Reverse	ACGAGACTCTGGCATGCTAACTAGT<br>CGCCACTTGTTCCTCTAAGAA
RPL13	Forward<br>Reverse	TCGTACGCTGTGAAGGCATC<br>GGTTGGTGTTCATCCGCTTG

Table S2. Eight histological and immunohistological parameters selected for quantification of Cartilage Repair as per International Cartilage Repair Society II Scoring System with Slight Modifications.

	Score for Different Attributes of Parameters (total Max Score 19)			
**Parameters**	**3**	**2**	**1**	**0**
Tissue Morphology	Hyaline	Mostly hyaline	Mixed hyaline/fibrocartilage	Fibrous tissue
Matrix Staining (Safranin O)	___	Normal Intensity	Reduced Intensity	No staining
Cell Distribution	___	Columnar	Columnar and Clusters	Clusters and disorganized
Surface Architecture	___	Smooth (>3/4)	Moderate (>1/2-3/4)	Severely irregular (<1/4)
Integration with subjacent bone	___	Full basal integration	Partial basal integration	No basal integration
Integration with subjacent cartilage	___	Full integration	Partial integration	No integration
Type I collagen immunostaining	None	Slight	Moderate	Abundant
Type II Collagen Immunostaining	Abundant	Moderate	Slight	None
